# News and Miscellany

**Published:** 1901-12

**Authors:** 


### News and Miscellany.

**The Alkaloidal Clinic** makes a very generous offer to new subscribers in a page advertisement appearing in this issue. Turn to their advertising page, Doctor, and read their proposition. It will be to your interest.

**New Buggies** sold to readers of the "Red Back" on the installment plan. See adv. of the Century Manufacturing Co.

---

**Medical Treatment of Cancer.**—Physicians interested in the successful treatment of cancer, as per article in the *Southern Medical Journal* for September, can obtain full information by addressing, with stamp, DR. H. B. ESMOND, West Fairlee, Vermont.

---

**The following Texas Doctors** registered at the New Orleans Polyclinic at the opening in November: Dr. D. D. Hamilton, Colmesneil; Dr. G. M. Jones, Rice's Crossing; Dr. J. F. Scruggs, Rogers' Prairie; Dr. L. Smith, Dallas; Dr. G. W. Vermillion, McDade; Dr. A. B. Kennedy, Bonham; Dr. A. M. Horner, Oakville.

---

**Good Location for a Physician** in a prosperous German community, railroad town, in central-southern Texas, can be secured by buying physician's residence. Price $2,700. The practice nets from $2,500 to $4,000 yearly. Terms $1,000 cash, balance on accommodating terms. Address RED, care TEXAS MEDICAL JOURNAL, Austin, Texas.

---

**Splendid Location** for a physician in rich and prosperous section of South Texas, can be secured by purchasing residence, office, stables, etc., and a cottage on separate lot. Practice realizes $3,500 to $6,000 yearly. Price of property, $3,200; $1,000 cash, balance on accommodating terms. Address F. W., care TEXAS MEDICAL JOURNAL, Austin, Texas.

---

**New Orleans Polyclinic** now in session, 15th year. Closes May 31, 1902.—Physicians will find the Polyclinic an excellent means for posting themselves upon modern progress in all branches of medicine and surgery. The specialties are fully taught, particularly laboratory work. For further information address Dr. Isadore Dyer, Secretary, New Orleans Polyclinic, postoffice box 797, New Orleans, La.

---

**Practice for Sale.**—A good home on acre lot, well improved in every way, with 5 acre pasture, conveniently located in a county seat, and railroad town 60 miles from Houston. Fine farming country, thickly settled with good Germans, where rice interest is just beginning to be develope . Terms reasonable. Property cost $4,000; goes for $3,500. Practice has averaged $3,000 for 20 years. Reason for selling is bad health. Write if you mean business, C. B., care Texas Medical Journal.

---

**Silver Wedding.** Mr. Reinhard Von Boeckmann, the head of the great printing house of Von Boeckmann, Schutze & Co., State Printers, Austin, Tex., and Mrs. Von Boeckmann celebrated the 25th anniversary to their wedding on the evening of November 4, (ult) at their handsome residence, San Jacinto and Eighteenth streets. On that occasion they received a host of attached friends who warmly congratulated them, and later sat down to a banquet where for two or three hours there was a flow of—beer, champagne and rhetoric. Dear old Ex-Governor Lubbock, the last but one, of the Confederate Government, honored the occasion with his presence, and was, as usual, very happy in his speech. Mr. and Mrs. Von Boeckmann were the recipients of loads of beautiful presents, as well as showers of congratulations and good wishes for many happy returns.

---

**The Confederate Reunion at Dallas,** and the reunion of the Army and Navy Medical Officers of the Confederate States will take place April 22 to 25th inclusive, instead of on the date before announced. Preparations are being made to accommodate half a million of people, and it will be the greatest, grandest and most interesting gathering since, well—the day of Pentecost—whenever and wherever that was. The finance committee of the Medicos want all contributions sent in by 1st of January. Dr. A. M. Elmore, chairman, Dallas, and "we'uns" want every doctor and son-of-a-doctor to *ante.* Send Dr. Elmore a ten, boys, right away. The spirit of sixty-one has descended to this generation, and sons of veterans must keep alive the memory of their sires, and the great principles for which they fought, bled and died—in vain.

